# S100A8 and S100A9 proteins form part of a paracrine feedback loop between pancreatic cancer cells and monocytes

**DOI:** 10.1186/s12885-018-5161-4

**Published:** 2018-12-17

**Authors:** Taoufik Nedjadi, Anthony Evans, Adnan Sheikh, Lawrence Barerra, Suliman Al-Ghamdi, Lucy Oldfield, W. Greenhalf, John P. Neoptolemos, Eithne Costello

**Affiliations:** 1 King Abdullah International Medical Research Centre, King Abdulaziz Medical City, Ministry of National Guard-Health Affairs, P. O. Box 9515, Jeddah, 21423 Saudi Arabia; 2 Department of Molecular and Clinical Cancer Medicine, Institute of Translational Medicine, The University of Liverpool, Liverpool, UK

**Keywords:** Pancreatic Cancer, Stroma, S100A8, S100A9, Cytokines, Monocytes

## Abstract

**Background:**

The secretion of soluble factors enables communication between tumour cells and the surrounding microenvironment and plays an important role in oncogenesis. Pancreatic ductal adenocarcinoma (PDAC) is characterised by a highly reactive microenvironment, harbouring a variety of cell types, including S100A8/S100A9-expressing monocytes. S100A8/S100A9 proteins regulate the behaviour of cancer cells by inducing pre-metastatic cascades associated with cancer spread. The aim of this study was to examine how S100A8/A9 proteins mediate tumour-stroma crosstalk in PDAC.

**Methods:**

Cytokine profiling of pancreatic cancer cell-derived conditioned media was performed using Bio-Plex Pro 27 Plex Human Cytokine assays. Protein expression and activation of downstream signalling effectors and NF-κB were assessed by western blotting analysis and reporter assays respectively.

**Results:**

Stimulation of cultured pancreatic cancer cells with S100A8 and S100A9 increased the secretion of the pro-inflammatory cytokines IL-8, TNF-α, and FGF. S100A8, but not S100A9 induced PDGF secretion. Conversely, pancreatic cancer cell-derived conditioned media and the individual cytokines, TNF-α and TGF-β induced the expression of S100A8 and S100A9 proteins in the HL-60 monocytic cell line and primary human monocytes, while FGF and IL-8 induced the expression of S100A9 only. S100A8 and S100A9 activated MAPK and NF-κB signalling in pancreatic cancer. This was partially mediated via activation of the receptor of advanced glycosylation end-product (RAGE).

**Conclusion:**

S100A8 and S100A9 proteins induce specific cytokine secretion from PDAC cells, which in turn enhances the expression of S100A8/A9. This paracrine crosstalk could have implications for PDAC invasiveness and metastatic potential.

**Electronic supplementary material:**

The online version of this article (10.1186/s12885-018-5161-4) contains supplementary material, which is available to authorized users.

## Background

Pancreatic ductal adenocarcinoma (PDAC) is an aggressive malignancy and a leading cause of cancer deaths worldwide [1]. Despite advances in our understanding of the pathophysiology of PDAC, there have been limited improvements in clinical outcome. This is in part due to the presence of a highly reactive stroma that promotes carcinogenesis, confers resistance to conventional therapy [2–5] and increases PDAC aggressiveness [6]. The tumour stroma comprises extracellular matrix and a variety of cell types including cancer-associated fibroblasts, stellate cells, endothelial cells and inflammatory cells such as macrophages and monocytes [7, 8]. The latter secrete the potent chemokines S100A8 and S100A9 which are low molecular proteins of S100 family of EF-hand calcium binding proteins. Both proteins are constitutively expressed in cells of myeloid origin such as monocytes and neutrophils [9]. Once secreted in the extracellular space, S100A8/A9 act as chemo-attractants recruiting further inflammatory cells and creating an inflammatory microenvironment that promotes tumour development [10, 11]. In-vivo and in-vitro experiments have shown a strong link between the levels of S100A8/S100A9 and several disorders such as cystic fibrosis, rheumatoid arthritis, atherosclerosis and cardiovascular disease [11–14]. Secreted S100A8/S100A9 proteins have also been implicated in cancer growth [15, 16] and in the establishment of a favourable environment for metastasis by promoting the migration of monocytes and tumor cells to metastatic sites [17, 18]. This process involves vascular endothelial growth factor-A (VEGF-A) and TGF-ß-SMAD4 signalling pathways and both toll-like receptor-4 (TLR-4) [19], and the receptor for advanced glycation end-products (RAGE) [20]. However little is known about S100A8 and S100A9-mediated cross talk between stromal monocytes and pancreatic cancer cells. In the current study, we show that exposing monocytes to pancreatic tumour cell-derived conditioned media increased the expression of both S100A8 and S100A9. Secreted S100A8/A9 from monocytes, in turn, led to secretion of several cytokines from pancreatic tumour cells. This was mediated by RAGE and was associated with phosphorylation of Erk1/2, p38 kinase and activation of the nuclear factor kappa-light-chain-enhancer of activated B cells pathway (NF-κB).

## Methods

MAPK antibodies (phospho-JNK, phospho-Erk1/2 and phospho-p38) were obtained from Cell Signaling Technology. S100A8 and S100A9 antibodies were purchased from Santa Cruz and the Bio-Plex Pro 27 Plex Human Cytokine, Chemokine and Growth Factor Assay was from Bio-Rad Laboratories Ltd., Hercules, CA, USA.

### Cell culture

Pancreatic cancer cell lines; CFPAC-1 (ATCC: CRL-1918), Suit-2, BxPC-3 (ATCC: CRL-1687) and Panc-1 (ATCC: CRL-1469) were obtained from ATCC (Rockville, MD) and maintained in a humidified incubator at 37 °C in RPMI-1640 containing 10% FBS, 2 mm l-glutamine, 2500 IU/mL penicillin and 5 μg/mL streptomycin (all from Sigma, Poole, UK). All cell lines were authenticated using short tandem repeat profiling against international reference standards.

### Isolation of human monocytes

Blood samples were obtained with written consent from two healthy volunteers under approved protocols at the Royal Liverpool University Hospital. Human monocytes were isolated from peripheral blood mononuclear cells (PBMCs) obtained from buffy coat preparations. Enriched monocyte fractions were isolated by positive selection using CD14-magnetic beads (Miltenyi Biotec, Bergisch Gladbach, Germany) following the manufacturer’s instructions. Monocytes were eluted in 5 mL MACS buffer and the purity (> 90%) verified by FACS analysis using anti-CD14 conjugated FITC antibody or isotype control.

### Stimulation of human monocytes

Freshly isolated monocytes were plated in 24-well plates in RPMI 1640 media supplemented with 10% FBS and stimulated with tumour cell-conditioned media from Panc-1, Suit-2 or BxPC-3 cell lines. Isolated monocytes were also treated with recombinant human growth factors (TGF-β, VEGF, IL-8 and FGF). After 24 h incubation, cell lysates were prepared using Tris/SDS lysis buffer and subjected to immuno-blotting assays.

### Conditioned media preparation

Pancreatic cancer cells were counted and seeded in 6-well plates in RPMI media. After 24 h, cells were washed twice with PBS and incubated in serum free-RPMI and treated with 2 μg/mL of the recombinant proteins S100A8-GST, S100A9-GST or GST and incubated for an additional 24 h at 37 °C. For blocking experiments, cells were incubated with anti-RAGE antibody (R&D, Abingdon, UK) for 1 h prior to addition of recombinant proteins. For neutralising experiments, S100A8-GST and S100A9-GST proteins were incubated with respective neutralising antibodies (anti-S100A8 and anti-S100A9) for 1 h at 37 °C before treating cancer cells in culture. Cell supernatants were collected, centrifuged at 500x g for 5 min, to remove cellular debris, and then filtered through 0.2 μm filter units. Conditioned media were stored in -80 °C freezer until use.

### Cytokines profiling assay

A Luminex assay (Bio-Plex Pro 27 Plex Human Cytokine kit) enabling the simultaneous measurement of 27 secreted cytokines in the conditioned media of pancreatic cancer cells was performed. Briefly, serially diluted standards and undiluted conditioned media (50 μL) were added to a microfilter plate containing antibody-coupled beads for each of the 27 analytes and incubated for 60 min with continuous shaking at room temperature. After washing steps, the biotinylated detection antibodies were added for 30 min with shaking. The microfilter plate was washed again, and Streptavidin-PE (50 μL) added and incubation continued at room temperature with shaking (900 rpm for 1 min followed by 300 rpm for 15 min). Assay buffer (125 μL) was added to each well of the microfilter plate before being read on a Bio-Plex 200 machine.

### Immunoblotting analysis

Total cell lysates were extraction by re-suspending cells in 100 mm Tris–HCl (pH 6.8) containing 2% *w*/*v* SDS and a protease inhibitor cocktail (Complete, Mini, EDTA-free protease inhibitors; Roche Applied Science, UK). Protein samples were quantified using a BCA protein assay kit (Perbio Science Ltd., Cramlington, UK) then subjected to SDS–PAGE. Separated proteins were transferred to hybond nitrocellulose membranes (Amersham Biosciences, Bucks, UK). Membranes were then blocked for 1 h with TBS containing 0.1% Tween-20 (TBS-T) and 5% milk (Bio-Rad Laboratories Ltd., Hemel Hempstead, Hertfordshire, UK) and then incubated overnight at 4 °C with antibodies specific for phospho-JNK, phospho-ERK1/2 and phospho-p38 (Cell Signaling Technology, Beverly,CA, USA) diluted 1:1000 in 5% BSA in TBST. The β-actin (clone AC-15, Sigma, Poole, UK) was used at a 1:10,000 dilution. Blots were washed with TBST and incubated for 1 h with horseradish-peroxidase (HRP)-conjugated secondary antibodies diluted 1:4000 in 5% skim milk in TBST. Bound HRP was visualised using the enhanced chemiluminescence kit (PerkinElmer Life Sciences, Bucks, UK).

### NF-κB luciferase assay

Pancreatic cancer cells, Panc-1, were trypsinised, counted and transfected using nucleofector and nucleofector solution-R (Amaxa, UK) according to the manufacturer’s instructions. Cells were transfected with 3 μg of NF-κB plasmid or control plasmid from Clontech. 3 μg of GFP plasmid (Amaxa) was also used to monitor transfection efficiency. Cells were immediately removed from cuvettes, transferred into prepared 6-well plates and incubated in a humidified 37 °C/ 5% CO_2_ incubator. After 24 h of incubation, the cells were washed twice with PBS and incubated in serum free RPMI with or without 10 μg/mL of polymyxin-b, the latter used in order to monitor the stimulatory effect of bacterial LPS. Cells were treated with 2 μg/mL of either S100A8, S100A9, GST recombinant proteins or human TNF-α (50 ng/mL), as a positive control of NF-κB induction, and incubated for an additional 24 h. Luciferase activity was measured according to the Clontech kit recommendations (Clontech, Mountain View, CA, USA).

### Statistical analysis

Statistical analyses were performed using student t-test. A *p*-value less than 0.05 was considered statistically significant.

## Results

### Cytokine profiling of pancreatic cancer cell-derived conditioned media

**Fig. 1:**
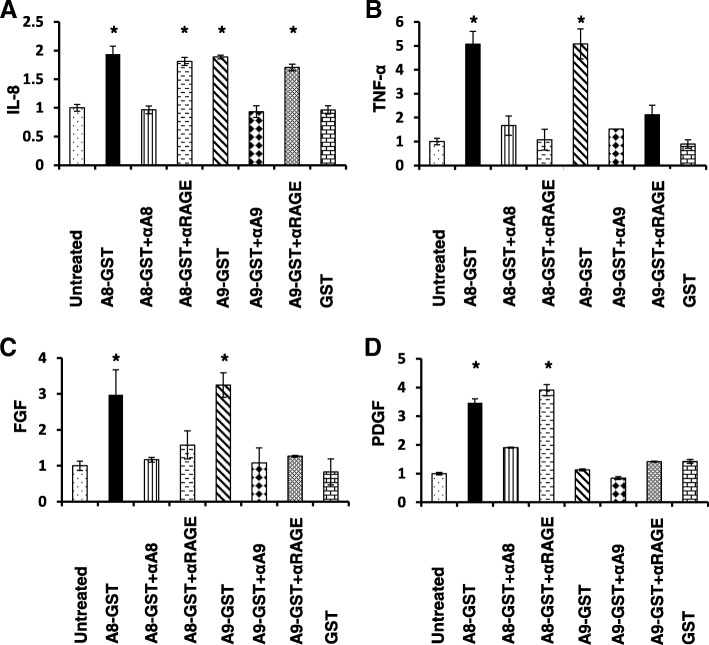
Effects of S100A8 and S100A9 proteins on the secretion of selected cytokines from Panc-1 cells. Conditioned media were collected from untreated cells or cells treated for 24 h with S100A8-GST (A8-GST), S100A9-GST (A9-GST), or treated with recombinant proteins which have been pre-incubated for 1 h with neutralising antibodies (A8-GST + αA8, A9-GST + αA9) or GST protein only. Conditioned media were also collected from cancer cells pre-treated for 1 h with anti-RAGE blocking antibody and then stimulated with recombinant proteins (A8-GST + αRAGE, A9-GST + αRAGE). The cytokine levels were measured using the Bio-Plex Pro 27 Plex Human Cytokine kit, and expressed relative to untreated control levels. Experiments were performed in duplicate three times and error bars denote standard error. * = *P* values < 0.05

Previously, we reported the presence of both S100A8-positive and S100A9-positive stromal monocytes in the tumour microenvironment of pancreatic and colorectal tumours [15]. Moreover, we observed that the number of S100A8-positive monocytes was significantly lower in tumours which lacked Smad-4 [21], suggesting a paracrine interaction between tumour cells and the monocytic stromal compartment. To decipher crosstalk between PDAC tumour cells and S100A8- and S100A9-expressing monocytes, we examined whether stimulation of Panc-1 pancreatic cancer cells with recombinant S100A8-GST or S100A9-GST fusion proteins altered their cytokine secretion, compared to stimulation with the control protein, GST. The profile of cytokines secreted from unstimulated Panc-1 cells is shown in Additional file 1: Table S1. Following incubation of Panc-1 cells with either S100A8-GST or S100A9-GST recombinant proteins, significant increases in IL-8, TNF-α and FGF were observed (Fig. 1a-c). Incubation of recombinant S100A8-GST and S100A9-GST proteins with respective anti-S100A8 and anti-S100A9 neutralising antibodies impaired their stimulatory effects, indicating that the stimulation was specific to S100A8 and S100A9. Moreover, incubation of Panc-1 cells with an anti-RAGE-blocking antibody prior to the addition of recombinant proteins reversed the stimulatory effects of S100A8 and S100A9 on TNF-α, and FGF, but not IL-8. Finally, the level of PDGF was increased after treatment of Panc-1 cells with recombinant S100A8 protein, but not recombinant S100A9 protein, and this increase was blocked by anti-S100A8– but not anti-RAGE– neutralising antibodies (Fig. 1d). Taken together, our data suggest that S100A8 and S100A9 have both overlapping and distinct effects on cytokine secretion, with those effects only partly mediated by the RAGE receptor.

### Conditioned media from pancreatic cancer cells induced S100A8/A9 expression in monocytes

**Fig. 2:**
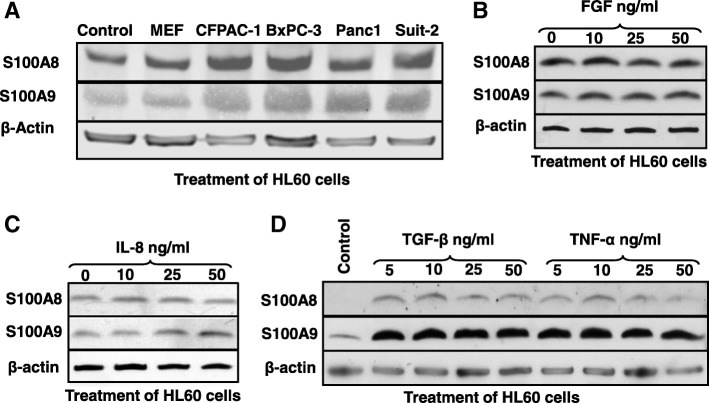
Effects of individual cytokines and secreted factors from pancreatic cancer cells on expression of S100A8 and S100A9 proteins in monocytes. A] Western blot analysis of S100A8 and S100A9 expression in HL-60 cells after treatment with conditioned media (CM) derived from the indicated pancreatic cancer cell lines; CFPAC-1, BxPC-3, Panc-1 and Suit-2, B, C and D] Western blot analysis of S100A8 and S100A9 expression in HL-60 cells following incubation with increasing concentrations of FGF, IL-8, TGF-β and TNF-α respectively. β-actin was used as a loading control

Having observed that S100A8 and S100A9 proteins altered the secreted cytokine profile from Panc-1 cells, we next asked whether secreted factors from Panc-1 and other PDAC cell lines could influence the expression of S100A8/A9 in monocytes. Treatment of the monocytic cell line HL-60 with conditioned media from CFPAC-1, BxPc-3, Panc-1 and Suit-2 led to a pronounced increase in the expression of both S100A8 and S100A9 (Fig. 2a). This effect was not observed when HL-60 cells were incubated with mouse embryonic fibroblast (MEF)-derived conditioned media or control unconditioned media (Fig. 2a).

### Cytokines induce expression of S100A8/A9 in monocytes

Having established that pancreatic cancer cells secrete factors that induce the expression of monocytic S100A8 and S100A9 (Fig. 2a) and that the S100 proteins themselves induced the secretion of FGF, IL-8, and TNF-α from pancreatic cancer cells (Fig. 1), we questioned whether FGF, IL-8 and TNF-α might contribute to the induction of monocytic S100A8 and S100A9 expression in a paracrine fashion. TGF-β was also examined, as it was previously shown to induce expression of S100A8/A9 in lung Mac 1^+^-myeloid cells [17]. Neither FGF nor IL-8 altered S100A8 in HL-60 cells (Fig. 2b and C), and only a very modest increase was observed for S100A9 expression after addition of either cytokine (Fig. 2b and c). Both S100A8 and S100A9 expression were increased in HL-60 cells (Fig. 2d) after the addition of recombinant human cytokines TGF-β and TNF-α, although a dose-dependent effect was not observed.

**Fig. 3:**
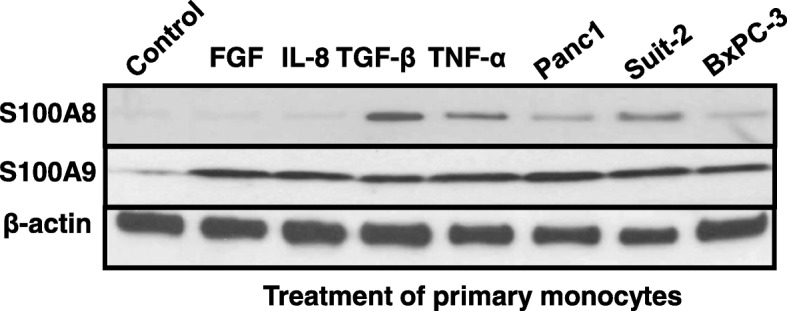
Effects of individual cytokines and secreted factors from pancreatic cancer cells on expression of S100A8 and S100A9 proteins in primary human monocytes. Western blot analysis of S100A8 and S100A9 expression in primary human monocytes after incubation with 10 ng/mL of the following cytokines FGF, IL-8, TGF-β and TNF-α, and after treatment with CM from Panc-1, Suit-2 and BxPC-3 cell lines

We next determined whether PDAC cell-conditioned media or cytokines could induce S100A8 and S100A9 expression in primary human monocytes. Conditioned media from Panc-1, Suit-2 and BxPc3 cancer cell lines caused increased expression of S100A8 and S100A9 proteins in primary human monocytes (Fig. 3). FGF and IL-8 treatment resulted in a strong induction of S100A9, but not S100A8 expression in primary human monocytes, while TGF-β and TNF-α both induced S100A8 and S100A9 expression in primary monocytes (Figs. 3).

### S100A8/A9 activate downstream signalling pathways

**Fig. 4:**
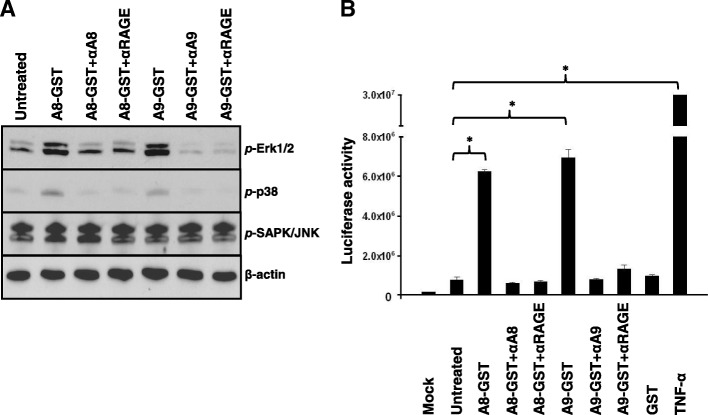
Signalling downstream of S100A8 and S100A9. A] Panc-1 cells were treated with [2 μg/mL] of recombinant A8-GST, A9-GST, A8-GST + αA8, A9-GST + αA9, A8-GST + αRAGE and A9-GST + αRAGE for 60 min and probed by Western blotting for the indicated phosphoproteins. β-actin served as a loading control. B] Panc-1 cells were transfected with NF-κB and control reporters [2 μg of DNA] and stimulated with recombinant A8-GST, A9-GST or GST [2 μg/mL] for 24 h. Cells treated with TNF-α [10 ng/mL] served as a positive control for NF-κB induction. For blocking conditions, cells were treated with anti-RAGE blocking antibody for 1 h prior to stimulation. S100A8-GST and S100A9-GST were incubated with anti-S100A8 and anti-S100A9 neutralising antibodies for 1 h before addition to cancer cell cultures. Luciferase activity was plotted as the mean ± SEM of three independent experiments, each performed in triplicate. Error bars represent standard error (* *P* < 0.05)

We next investigated downstream mediators of S100A8 and S100A9 signalling in pancreatic cancer cells, including phospho-MAPK proteins (Fig. 4a) and NF-κB (Fig. 4b). Panc-1 cells treated with S100A8 and S100A9 proteins for one hour showed higher than control levels of the phospho-MAPK proteins, phospho-p38 and phospho-Erk-1/2 (Fig. 4a). These effects were respectively abrogated by pre-treatment of proteins with anti-S100A8 or anti-S100A9 antibodies. Moreover, this observation was RAGE-dependent, since blockage of RAGE receptor using anti-RAGE antibody was accompanied by lower levels of p-p38 and p-Erk-1/2 (Fig. 4a). The addition of S100A8/A9 recombinant proteins did not alter the phosphorylation status of SAPK/JNK. The effects of treatment of Panc-1 cells with S100A8 and S100A9 on the basal levels of these MAPK proteins was not examined. In this system, we conclude that treatment with S100A8 and S100A9 proteins is accompanied by higher levels of phosphorylated p38 and Erk1/2 than untreated controls, and that the RAGE receptor is required for this effect.

Ligand binding to RAGE receptor triggers intracellular signalling and stimulates a number of transcription factors including NF-κB [22]. Transient transfection of Panc-1 cells with an NF-κB reporter plasmid followed by incubation with recombinant S100A8 and S100A9 proteins for 24 h produced a significant activation of NF-κB luciferase activity compared to mock transfected, transfected but untreated or GST treated controls (Fig. 4b). The observed increase in luciferase activity was blocked by the relevant S100 antibodies. Moreover, it was exclusively related to the action of S100A8/A9 proteins and not to bacterial lipopolysaccharides (LPS) because addition of 10 μg/mL polymyxin-b, which is a potent inhibitor of LPS, gave similar results (Additional file 2: Figure S1). Pre-incubation with RAGE blocking antibody abolished the S100A8 and S100A9-induced NF-κB activation, indicating that S100A8 and S100A9 proteins activated NF-κB via the RAGE receptor. As expected, TNF-α which served as a positive control in this experiment, significantly promoted NF-κB luciferase activity.

## Discussion

The complex interaction between pancreatic tumour cells and surrounding immune cells is increasingly understood to be vital in sustaining tumour growth and progression [23, 24]. Stromal cells potentiate tumour development through the secretion of a complex network of autocrine and paracrine factors including cytokines, proteases and growth factors [25, 26].

We previously showed that the stromal compartment of colorectal and pancreatic cancers contain CD14^+^ monocytic cells expressing S100A8 and S100A9 proteins [21]. Moreover, we observed that Smad4-negative pancreatic and colorectal tumours contain fewer stromal S100A8-positive monocytes than their Smad4-positive counterparts [21, 27], suggesting a regulated relationship between cancer cells and surrounding monocytic cells. No such relationship was observed for S100A9-positive monocytes, indicating a distinction between S100A8- and S100A9-expressing tumour-associated monocytes. We also reported that exogenously added S100A8 and S100A9 proteins enhanced the migration and proliferation of colorectal and pancreatic cancer cells in culture [15]. In the current study, we found that S100A8 and S100A9 proteins influenced cytokine secretion from pancreatic cancer cells in overlapping but also distinct ways. Whilst both recombinant S100A8-GST and S100A9-GST proteins induced increased secretion of the pro-inflammatory cytokines IL-8 and TNF-α and the growth factor FGF, only S100A8-GST induced PDGF secretion. This again highlights a distinction between these two closely related S100 proteins.

S100A8/A9 are reported to stimulate cells through binding to the cellular receptor RAGE, a primary receptor which, when activated, is associated with amplified inflammatory conditions and tumour progression [27]. Here we found that the secretion of TNF-α and FGF was mediated via the RAGE receptor, whilst the RAGE receptor was not required for IL-8 and PDGF secretion. S100A8/A9 signaling from receptors other than the RAGE receptor has previously been reported, including the binding of S100A8/A9 to Toll-like receptor-4 receptor and the newly discovered EMMPRIN receptor that exclusively binds to S100A9 but not S100A8 [28, 29].

Previous studies have elucidated a role for S100A8/A9 in preparing a pre-metastatic niche in distant organs through activation of serum amyloid A3 and toll-like receptor 4 (TLR4) [17, 19]. For patients with pancreatic cancer, release of S100A8/A9 proteins from myeloid cells increases CD33^+^CD14^−^HLA-DR^−^ myeloid-derived suppressor cells [30]. Our observation that media conditioned from pancreatic cancer cells, as well as the individual cytokines TGF-β and TNF-α, induced the expression of S100A8 and S100A9 proteins in HL-60 cells and primary monocytes supports the concept of a paracrine feedback loop, whereby cancer cells induce monocytic S100A8/A9 expression through cytokine secretion and the S100 proteins in turn induce cytokine secretion. Secretion of TGF-β by pancreatic cancer cells induces extra-cellular matrix (ECM) formation and enhances fibrosis [31]. Both TGF-β and TNF-α have been associated with increased cancer cell proliferation and motility. Additionally, both TGF-β and TNF-α participate in the creation of inflammatory environments that enhance tumorigenesis and immunosuppressive activity blocking the host’s anti-tumour response [32]. Induction of S100A8 and S100A9 expression in response to primary lung tumor cell-derived soluble factors VEGF-A, TGF-β and TNF-α in myeloid cells prior to tumor metastasis has been reported [17].

Binding of S100A8 and S100A9 to RAGE receptor activates a cascade of downstream signalling pathways [27]. We report here a RAGE-dependent increase in levels of phosphor-Erk1/Erk2 and phospho-p38 phosphorylation following S100A8 and S100A9 treatment of pancreatic cancer cells, although we did not examine the basal levels of these proteins. Furthermore, the S100 proteins activated the NF-κB pathway through RAGE. Activation of the MAPK/NF-κB signaling axis is known to enhance tumor growth, survival, and migration [33]. Moreover, NF-κB activation is also critical in establishing an inflammatory microenvironment which in turn sets the stage for cancer progression [33].

In summary, our data suggest the existence of paracrine feedback loop between stroma-associated S100A8/A9-secreting cells and pancreatic tumour cells. This feedback loop may affect tumour progression. Hence, targeting S100A8/A9 represents a potential therapeutic option to curtail the aggressive nature of pancreatic cancer.

## Additional files

Additional file 1: **Table S1.** Comparison of levels of 27 cytokines in conditioned media from pancreatic cancer cells. Panc-1 cells were cultured in serum-free media and treated with recombinant proteins for S100A8-GST, S100A9-GST and GST. S100A8-GST and S100A9-GST were incubated with respective neutralizing antibodies (anti-S100A8 and anti-S100A9) for 1 h at 37 °C before addition to cancer cells. For blocking experiments, cells were incubated with anti-RAGE antibody (80 μg/ml, R&D, UK) for 1 h prior to addition of recombinant proteins. Supernatants were collected 24 h later and analyzed using Bio-Plex Pro 27 Plex Human Cytokine kit. The results are presented as a mean of three independent experiments performed in duplicate (*p* < 0.05. NS: non-significant). (DOCX 21 kb)

Additional file 2: **Figure S1.** Specificity of signalling downstream of S100A8 and S100A9. Panc-1 cells were left untreated or incubated in the presence of polymyxin-b (10 μg/mL). The cells were transfected with NF-κB and control reporters [2 μg of DNA] and stimulated with recombinant A8-GST, A9-GST or GST [2 μg/mL] for 24 h. Cells treated with TNF-α [10 ng/mL] served as a positive control for NF-κB induction. For blocking conditions, cells were treated with anti-RAGE blocking antibody for 1 h prior to stimulation. S100A8-GST and S100A9-GST were incubated with anti-S100A8 and anti-S100A9 neutralising antibodies for 1 h before addition to cancer cell cultures. Luciferase activity was plotted as the mean ± SEM of three independent experiments performed in triplicate. Error bars represent standard error (* *P* < 0.05). (PPTX 53 kb)

## Abbreviations

FGF: Fibroblast growth factor
GST: Glutathione S-transferase
IL-8: Interleukin-8
LPS: Lipopolysaccharides
NF-κB: Nuclear factor kappa-light-chain-enhancer of activated B cells
PDAC: Pancreatic ductal adenocarcinoma
RAGE: receptor of advanced glycosylation end-product
TGF-β: Transforming growth factor- beta.
TLR-4: Toll-like receptor-4
TNF-α: Tumour necrosis factor-alpha
VEGF: Vascular endothelial growth factor
