# Supplementary material for: S100A8 and S100A9 proteins form part of a paracrine feedback loop between pancreatic cancer cells and monocytes
Source: BMC Cancer. 2018 Dec 17;18:1255. doi: 10.1186/s12885-018-5161-4 (PMC6296088; doi:10.1186/s12885-018-5161-4)
Supplement: Supplementary file 1 — Table S1. Comparison of levels of 27 cytokines in conditioned media from pancreatic cancer cells. Panc-1 cells were cultured in serum-free media and treated with recombinant proteins for S100A8-GST, S100A9-GST and GST. S100A8-GST and S100A9-GST were incubated with respective neutralizing antibodies (anti-S100A8 and anti-S100A9) for 1 h at 37 °C before addition to cancer cells. For blocking experiments, cells were incubated with anti-RAGE antibody (80 μg/ml, R&D, UK) for 1 h prior to addition of recombinant proteins. Supernatants were collected 24 h later and analyzed using Bio-Plex Pro 27 Plex Human Cytokine kit. The results are presented as a mean of three independent experiments performed in duplicate (p < 0.05. NS: non-significant). (DOCX 21 kb) [file 12885_2018_5161_MOESM1_ESM.docx]

| **Cytokines**  **Conc (ng/ml)** | **Control** | **A8-GST**  **(*p* value)** | **A8-GST+αA8**  **(*p* value)** | **A8-GST+αRAGE**  **(*p* value)** | **A9-GST**  **(*p* value)** | **A9-GST+αA9**  **(*p* value)** | **A9-GST+αRAGE**  **(*p* value)** | **GST**  **(*p* value)** |
| --- | --- | --- | --- | --- | --- | --- | --- | --- |
| IL-1b | 0.92 | 0.8  (*NS*) | 0.915  (*NS*) | 1  (*NS*) | 0.855  (*NS*) | 0.94  (*NS*) | 0.88  (*NS*) | 0.91  (*NS*) |
| IL-5 | 1.755 | 1.825  (*NS*) | 1.53  (*NS*) | 1.815  (*NS*) | 1.82  (*NS*) | 1.35  (*NS*) | 1.785  (*NS*) | 1.41  (*NS*) |
| IL-6 | 8.16 | 8.62  (*NS*) | 7.325  (*NS*) | 9.8  (*NS*) | 7.955  (*NS*) | 7.48  (*NS*) | 9.15  (*NS*) | 7.565  (*NS*) |
| IL-7 | 55.24 | 54.6  (*NS*) | 57.355  (*NS*) | 60.64  (*NS*) | 53.82  (*NS*) | 53.835  (*NS*) | 57.18  (*NS*) | 55.25  (*NS*) |
| IL-8 | 264.965 | 455.505  (*p=0.005*) | 256.7  (*NS*) | 475.43  (*p=0.015*) | 456.665  (*p=0.005*) | 246.315  (*NS*) | 443.18  (*p=0.017*) | 247.11  (*NS*) |
| IL-9 | 88.745 | 108.795  (*NS*) | 99.715  (*NS*) | 102.635  (*NS*) | 106.05  (*NS*) | 102.22  (*NS*) | 96.43  (*NS*) | 83.205  (*NS*) |
| IL-10 | 22.29 | 27.97  (*NS*) | 26.66  (*NS*) | 28.29  (*NS*) | 26.72  (*NS*) | 24.52  (*NS*) | 26.75  (*NS*) | 26.25  (*NS*) |
| IL-12(p70) | 185.425 | 210.725  (*NS*) | 204.9  (*NS*) | 205.86  (*NS*) | 194.81  (*NS*) | 218.165  (*NS*) | 195.025  (*NS*) | 184.42  (*NS*) |
| IL-13 | 24.005 | 23.745  (*NS*) | 26.795  (*NS*) | 24.27  (*NS*) | 24.94  (*NS*) | 26.745  (*NS*) | 26.87  (*NS*) | 26.3  (*NS*) |
| IL-15 | 12.23 | 13.12  (*NS*) | 11.825  (*NS*) | 14.005  (*NS*) | 13.15  (*NS*) | 11.43  (*NS*) | 13.09  (*NS*) | 10.745  (*NS*) |
| Eotaxin | 107.23 | 117.19  (*NS*) | 115.75  (*NS*) | 120.945  (*NS*) | 118.665  (*NS*) | 109.965  (*NS*) | 118.73  (*NS*) | 108.56  (*NS*) |
| PDGF-bb | 294.83 | 1045.635  (*p=0.02*) | 679.59  (*NS*) | 1249.535  (*p* =0.04) | 357.25  (*NS*) | 295.045  (*NS*) | 472.31  (*NS*) | 465.08  (*NS*) |
| FGF basic | 10.14 | 20.785  (*p=0.007*) | 11.76  (*NS*) | 15.78  (*p=0.051*) | 24.9  (*p=0.0007*) | 10.88  (*NS*) | 18.855  (*p=0.04*) | 9.4  (*NS*) |
| IFN-g | 85.225 | 98.945  (*NS*) | 104.195  (*NS*) | 101.76  (*NS*) | 78.035  (*NS*) | 91.86  (*NS*) | 107.585  (*NS*) | 101.305  (*NS*) |
| IP-10 | 103.12 | 117.375  (*NS*) | 126.22  (*NS*) | 141.98  (*NS*) | 107.68  (*NS*) | 96.85  (*NS*) | 108.925  (*NS*) | 100.445  (*NS*) |
| MIP-1a | 16.4 | 19.025  (*NS*) | 13.385  (*NS*) | 19.305  (*NS*) | 13.01  (*NS*) | 13.115  (*NS*) | 16.825  (*NS*) | 15.75  (*NS*) |
| RANTES | 1658.895 | 1537.885  (*NS*) | 1428.43  (*NS*) | 1783.145  (*NS*) | 1590.445  (*NS*) | 1481.55  (*NS*) | 1659.145  (*NS*) | 1738.17  (*NS*) |
| TNF-a | 1.875 | 10.755  (*p=0.03*) | 2.795  (*NS*) | 2.14  (*NS*) | 10.805  (*p=0.01*) | 2.105  (*NS*) | 3.91  (*NS*) | 1.08  (*NS*) |
| VEGF | 2286.45 | 2308.315  (*NS*) | 2597.2  (*NS*) | 2658.215  (*NS*) | 2339.92  (*NS*) | 2495.285  (*NS*) | 2241.195  (*NS*) | 2261.155  (*NS*) |

**Table S1:** Comparison of cytokines levels in conditioned media from pancreatic cancer cells. Panc-1 cells were cultured in serum-free media for 24h and treated with recombinant proteins for S100A8-GST, S100A9-GST and GST with or without neutralising antibodies. Where neutralising antibodies were used, S100A8-GST and S100A9-GST were incubated with respective neutralizing antibodies (anti-S100A8 and anti-S100A9) for 1 h at 37^0^C before treatment of cancer cells. For blocking experiments, cells were incubated with anti-RAGE antibody (80 µg/ml, R&D, UK) for 1h prior to addition of recombinant proteins. Supernatants were collected 24h later and analyzed using Bio-Plex Pro 27 Plex Human Cytokine kit. The results are presented as a mean of three independent experiments performed in duplicate (NS = non-significant).
