# Supplementary material for: S100A8 and S100A9 proteins form part of a paracrine feedback loop between pancreatic cancer cells and monocytes
Source: BMC Cancer. 2018 Dec 17;18:1255. doi: 10.1186/s12885-018-5161-4 (PMC6296088; doi:10.1186/s12885-018-5161-4)
Supplement: Supplementary file 2 — Figure S1. Specificity of signalling downstream of S100A8 and S100A9. Panc-1 cells were left untreated or incubated in the presence of polymyxin-b (10 μg/mL). The cells were transfected with NF-κB and control reporters [2 μg of DNA] and stimulated with recombinant A8-GST, A9-GST or GST [2 μg/mL] for 24 h. Cells treated with TNF-α [10 ng/mL] served as a positive control for NF-κB induction. For blocking conditions, cells were treated with anti-RAGE blocking antibody for 1 h prior to stimulation. S100A8-GST and S100A9-GST were incubated with anti-S100A8 and anti-S100A9 neutralising antibodies for 1 h before addition to cancer cell cultures. Luciferase activity was plotted as the mean ± SEM of three independent experiments performed in triplicate. Error bars represent standard error (* P < 0.05). (PPTX 53 kb) [file 12885_2018_5161_MOESM2_ESM.pptx]

## Slide 1
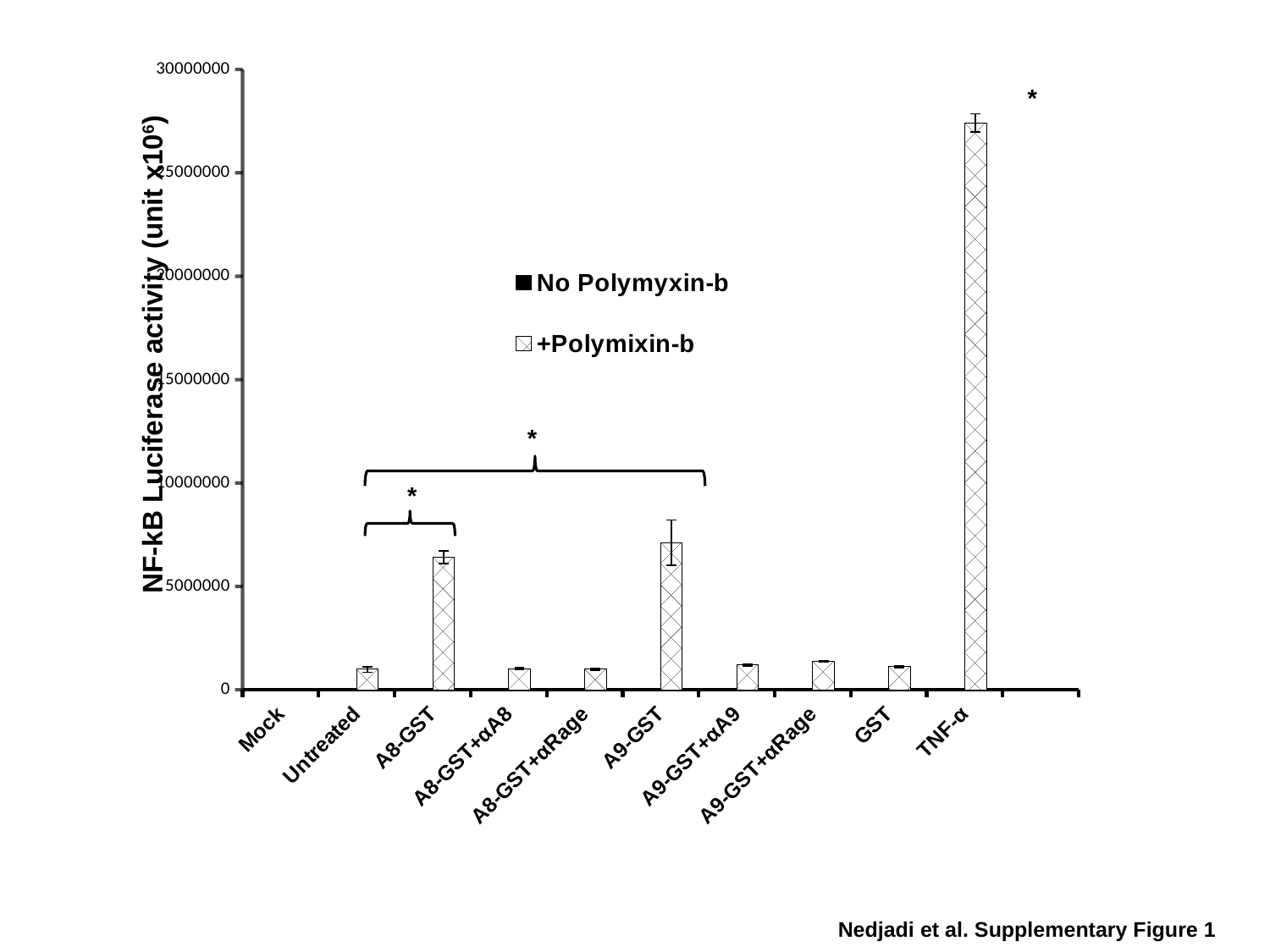

### Chart
| Category | | |
|---|---|---|
| Mock | 23034.666666666668 | 23034.666666666668 |
| Untreated | 783017.0 | 986072.6666666666 |
| A8-GST | 6253326.333333333 | 6404933.0 |
| A8-GST+αA8 | 856415.6666666666 | 1018410.6666666666 |
| A8-GST+αRage | 823245.0 | 989505.6666666666 |
| A9-GST | 6948047.333333333 | 7121180.333333333 |
| A9-GST+αA9 | 1046441.6666666666 | 1208851.6666666667 |
| A9-GST+αRage | 1341278.3333333333 | 1368236.6666666667 |
| GST | 996655.3333333334 | 1113708.0 |
| TNF-α | 26657061.333333332 | 27421590.666666668 |*
NF-kB Luciferase activity (unit x106)
*
Nedjadi et al. Supplementary Figure 1
